# Brain Gadolinium Biodistribution, Clearance, and Retention Following High-Dose Contrast-Agent Exposure in Mice: A Multimodal Study

**DOI:** 10.3390/toxics14100907

**Published:** 2026-10-14

**Authors:** Alon Zahavi, Stephen Richard, Olga Girshevitz, Basel Obied, Yoav Vardizer, Hila Kreizman-Shefer, Jacob Bajar, Judith Luckman, Dror Fixler, Nitza Goldenberg-Cohen

**Affiliations:** 1 Department of Ophthalmology, Rabin Medical Center, Beilinson Hospital, Petach Tikva 4941492, Israel; alonzahavi@tauex.tau.ac.il; 2 Gray Faculty of Medical and Health Sciences, Tel Aviv University, Tel Aviv 6997801, Israel; 3 Laboratory of Eye Research, Felsenstein Medical Research Center, Petach Tikva 4941492, Israel; 4 The Krieger Eye Research Laboratory, Bruce and Ruth Faculty of Medicine, Technion Institute of Technology, Haifa 3339419, Israel; steverit11@gmail.com (S.R.); basel.obied01@gmail.com (B.O.); 5 Faculty of Engineering and Institute of Nanotechnology & Advanced Materials, Bar-Ilan University, Ramat Gan 5290002, Israel; olga.girshevitz@biu.ac.il; 6 Department of Ophthalmology, Bnai-Zion Medical Center, Haifa 3339419, Israel; yoavardizer@gmail.com; 7 Department of Pathology, Bnai-Zion Medical Center, Haifa 3339419, Israel; hila.shefer@b-zion.org.il (H.K.-S.); jacob.bejar@gmail.com (J.B.); 8 Department of Radiology, Rabin Medical Center, Beilinson Hospital, Petach Tikva 4941492, Israel; judluc@msn.com; 9 School of Biomedical Engineering, Tel Aviv University, Tel Aviv 6997801, Israel; fixeled@tauex.tau.ac.il

**Keywords:** gadolinium-based contrast agents (GBCAs), gadolinium retention, brain deposition, particle-induced X-ray emission (PIXE), magnetic resonance imaging (MRI)

## Abstract

We evaluated gadolinium biodistribution, clearance, and long-term brain retention following high-dose gadolinium-based contrast agent (GBCA) exposure in 90 mice. Macrocyclic and linear agents were assessed using particle-induced X-ray emission (PIXE) alongside complementary elemental, imaging, and histological methods. Following intravenous administration, gadolinium was detected in blood and multiple tissues during the acute phase, with declining blood levels and detectable urinary excretion. No brain Gd signal was detected by PIXE at the long-term endpoints following single exposure. Repeated-exposure findings were descriptive because the individual subgroups contained only 1–3 animals. Endpoint MRI showed no apparent persistent signal changes, and H&E examination showed preserved tissue morphology. Metallothionein staining varied qualitatively, whereas Iba1 staining showed no apparent increase in microglial activation. Intraperitoneal administration produced no detectable blood gadolinium at the 1-h sampling timepoint. These findings characterize high-dose experimental exposure and support the feasibility of multimodal assessment of gadolinium biodistribution. Negative findings do not exclude residual Gd below the detection capabilities of the methods used.

## 1. Introduction

Gadolinium (Gd)-based contrast agents (GBCAs) have been widely used since 1988 to enhance the quality of magnetic resonance imaging (MRI) [1]. Once injected, GBCAs are rapidly distributed and primarily eliminated via renal and hepatobiliary pathways [1]. Reported safety concerns include systemic toxicity in patients with renal impairment, nephrogenic systemic fibrosis, encephalopathy, and prolonged contrast enhancement in cerebrospinal fluid [2,3,4,5,6,7,8].

GBCAs are generally considered safe. However, accumulating evidence suggests that trace amounts of Gd may be deposited in human tissues, including brain, bone, and kidneys, particularly following repeated exposure [9,10,11,12,13]. Earlier studies used neutron activation analysis to quantify rare-earth elements in human brain tissue and kidney stones [14,15], although these findings did not establish a link to GBCA exposure. Animal studies have examined GBCA-associated tissue distribution, retention, and potential toxicity, with findings varying by agent and exposure regimen [11,16,17,18,19,20,21,22]. The clinical significance of Gd retention remains uncertain, and histopathological confirmation in humans is limited [4,10,12,13,23]. Symptoms attributed to gadolinium exposure have also been described in a case series of physicians with self-diagnosed gadolinium deposition disease [24], although the evidence for a causal relationship remains uncertain [23]. The contrasting ligand structures and stability profiles of gadoterate and gadobenate provide a rationale for comparing their biodistribution and tissue retention [1,25]. In addition, alternative administration routes may affect biodistribution and clearance. Gd retention in multiple organs has also been investigated after intra-articular GBCA administration in rats, illustrating the relevance of administration route when interpreting tissue distribution [26].

The two agents evaluated in this study, gadoterate meglumine (Dotarem) and gadobenate dimeglumine (MultiHance), enhance MRI contrast by shortening proton relaxation times, improving the visualization of lesions with blood–brain barrier disruption or abnormal vascularity. Dotarem is an ionic macrocyclic agent in which Gd^3+^ is coordinated by a ring-shaped DOTA ligand. MultiHance is an ionic linear agent in which Gd^3+^ is coordinated by an open-chain, benzyloxy-substituted DTPA-derived ligand. Both agents are formulated as meglumine salts. MultiHance exhibits weak, transient interactions with serum proteins that increase its relaxivity and may improve lesion conspicuity [1].

Distinguishing transient tissue exposure from persistent retention is important for interpreting potential Gd-associated toxicity. Measurements in blood describe circulating exposure, whereas urinary measurements and assessments of kidney, liver, and other tissues provide complementary information about elimination and tissue distribution. Evaluating brain tissue at later timepoints addresses whether Gd remains detectable after circulating levels have declined. Combining elemental measurements with assessments of tissue morphology and cellular responses allows retention findings to be considered alongside potential biological effects.

Particle-induced X-ray emission (PIXE) enables spatially resolved elemental mapping and quantification at trace concentrations in tissue sections [27,28,29]. Although ICP-MS provides greater analytical sensitivity and is widely regarded as the reference method for bulk Gd quantification, conventional ICP-MS requires tissue digestion and therefore does not preserve spatial information. PIXE offers the complementary advantage of mapping elemental distribution directly within intact tissue sections, with detection typically in the parts-per-million range. Nuclear microscopy incorporating PIXE has previously been used to quantify Gd distribution in biological tissue sections following GBCA administration [29]. Micro-PIXE has also demonstrated heterogeneous distributions of Gd nanoparticles within human glioblastoma xenografts in mice [30]. However, to our knowledge, PIXE has not previously been applied to brain sections specifically to evaluate long-term Gd retention.

Other biological applications include microPIXE analysis of elemental composition in proteins [31]. Tear-fluid assessment requires attention to collection conditions, including Schirmer-strip standardization [32], and PIXE has been used to characterize trace elements in human tears [33]. Complementary approaches include X-ray absorption spectroscopy for investigating elemental chemical environments in biological samples [34] and ICP-based methods for multielement profiling, including applications to geochemical and environmental samples [35].

This study was designed to characterize Gd biodistribution and clearance following high-dose IV GBCA administration in mice and to evaluate whether Gd remained detectable in brain tissue at long-term endpoints. A multimodal approach was selected to address complementary analytical and biological questions. PIXE assessed elemental Gd in blood, body fluids, and tissue sections, while SEM-EDS provided localized elemental assessment in selected brain regions. Endpoint MRI evaluated persistent brain signal changes, H&E examination assessed tissue morphology, and metallothionein and Iba1 staining assessed metal-associated protein expression and microglial responses, respectively. This design placed the brain retention findings within the broader pattern of systemic distribution and elimination while examining tissue responses relevant to potential toxicity. The study also evaluated the applicability of PIXE within this analytical framework. Both macrocyclic and linear agents were examined, and a separate exploratory IP protocol assessed whether this administration route produced detectable systemic exposure under the sampling conditions evaluated.

## 2. Materials and Methods

A total of 90 male C57BL/6 wild-type mice aged 6 to 8 weeks were obtained from ENVIGO Laboratories (Ness Ziona, Israel). Animals were maintained under standard laboratory conditions and handled in accordance with the ARVO Statement for the Use of Animals in Ophthalmic and Vision Research, the National Institutes of Health guidelines, and the ARRIVE guidelines. All experimental procedures conducted in this study were approved by the Animal Care and Use Committee of Rabin Medical Center (approval #022_b16473_13).

The standard clinical dose of GBCAs for MRI is 0.1 mmol/kg [1]. High doses were selected to create substantial gadolinium exposure and increase the likelihood of detecting its distribution across blood and tissues using the selected analytical methods. This design enabled assessment of whether detectable brain gadolinium persisted after a high-dose challenge and evaluation of the applicability of PIXE within a multimodal analytical framework. The IV dosing was informed by previous rodent studies using 2.5 mmol Gd/kg per injection [17].

Two GBCAs were used in this study: the macrocyclic agent Dotarem (gadoterate meglumine, 0.5 mmol/mL; Guerbet, Villepinte, France) and the linear agent MultiHance (gadobenate dimeglumine, 0.5 mmol/mL; Bracco Imaging, Milan, Italy). Both agents were administered IV at a fixed volume of 0.12 mL, corresponding to 0.06 mmol Gd per mouse. Dotarem was also administered IP at a fixed volume of 0.20 mL, corresponding to 0.10 mmol Gd per mouse. Assuming a typical body-weight range of 20–25 g for male C57BL/6 mice aged 6–8 weeks, the estimated doses were 2.4–3.0 mmol Gd/kg IV and 4.0–5.0 mmol Gd/kg IP. On a mmol/kg basis, the estimated IV and IP doses were approximately 24–30 and 40–50 times the standard clinical dose of 0.1 mmol/kg, respectively. The term “high-dose” therefore refers to administered doses substantially exceeding standard clinical dosing. The IP protocol was a separate exploratory component designed to assess whether this administration route produced detectable systemic Gd exposure in mice. IV administration corresponds to the clinical route used for Dotarem-enhanced MRI.

Animals were anesthetized by intraperitoneal injection of ketamine (80 mg/kg) and xylazine (4 mg/kg) before contrast administration to ensure immobilization during the injection procedure. The GBCA (Dotarem or MultiHance) or saline was administered according to group allocation. IV injections were performed via the tail vein, and IP injections were performed using standard techniques. At the designated sampling timepoint, blood was collected by cardiac aspiration under deep anesthesia. The animals were subsequently euthanized by cervical dislocation, after which the brain and other organs were harvested.

The study comprised four experimental components: (1) biodistribution and clearance following a single IV Dotarem injection, (2) long-term retention following a single IV injection of Dotarem or MultiHance, (3) repeated IV Dotarem exposure, and (4) assessment of systemic exposure following repeated IP administration of Dotarem, with saline-treated controls. Group allocation, dosing regimens, and euthanasia timepoints are summarized in Supplementary Table S1. Tissues and body fluids were collected at predefined timepoints for multimodal analysis.

The biological control group comprised 16 IP saline-injected mice: eight received two injections 3 days apart, and eight received two injections 10 days apart. These animals provided control specimens for the study’s analytical assessments. Supplementary Table S1 reports the control-group allocation.

Blood, urine, tear, and tissue samples were collected at predefined timepoints for elemental and histological analyses. Tissues included brain, eye, liver, kidney, spleen, bone, and skin. The numbers of animals contributing samples to each analytical method are reported in Supplementary Table S1. Analyses were performed on the specimens available and suitable for each method. Representative images are shown in the figures, whereas overall conclusions were based on all evaluable samples.

Brains were collected from five Dotarem and five MultiHance mice euthanized at 28 days after Gd administration, and ten saline controls. Samples were immersed in Fomblin perfluoropolyether solution (Solvay, Italy) and imaged with a 9.4 T horizontal MRI system (Bruker Biospec, Ettlingen, Germany). A 3D T1-weighted fast low-angle shot (FLASH) sequence was used, with TR/TE = 15/4.7 ms and a flip angle of 15°. The field of view measured 19.2 × 19.2 × 15.5 mm^3^ and matrix size was 192 × 192 × 31, resulting in an in-plane resolution of 100 μm. The number of averages was 4, and total acquisition time was 6 min.

MRI findings were compared with brains from IP saline-injected control mice. Data were processed using Medical Image Processing, Analysis and Visualization (MIPAV) software version 11.3.3 (NIH; http://mipav.cit.nih.gov, accessed on 13 January 2022). Following MRI acquisition, brains were sectioned for subsequent histological analyses.

Samples were analyzed with a 1.023 MeV proton beam generated by a 1.7 MV tandem Pelletron accelerator, model 5SDH (National Electrostatics Corp., Middleton, WI, USA). Brain slices of 50–100 µm covering the hippocampus region were fixed in 4% paraformaldehyde. The PIXE data were analyzed using GUPIX software (https://www.physics.uoguelph.ca/gupix-software-download, accessed on 31 August 2026) version 2.2.4, under the assumption that the targets (specifically, the measurable areas) were thin and homogeneous. GUPIX performs least-squares fitting of PIXE spectra followed by conversion of peak areas into elemental concentrations (in ng/cm^3^) using a standardization technique involving fundamental parameters and predetermined instrumental constants. The spectrum model was built from the relevant X-ray peaks and then digitally filtered, and the results were compared with the digitally filtered measured spectrum.

PIXE quantifies elemental concentrations but does not distinguish between chemical forms. Reference samples were prepared by applying 30 µL of Dotarem or MultiHance solutions at concentrations of 0.005–0.5 mM onto filter paper to assess Gd detection under these experimental conditions. This assessment did not establish a tissue-specific detection limit, as validation using biological tissues containing known Gd concentrations was not performed.

Tissue samples were fixed for 2 h in Karnovsky fixative [2.5% glutaraldehyde with 2.5% paraformaldehyde in 0.1 M sodium cacodylate buffer (pH 7.4)] at room temperature, placed in a cold room (4 °C) overnight, and then washed with 0.1 M sodium cacodylate buffer. The samples were postfixed in 1% OsO_4_, 0.5% K_2_Cr_2_O_7_, and 0.5% K4 [Fe(CN)_6_] in 0.1 M cacodylate buffer (pH 7.4) for 1 h at room temperature, washed twice with 0.1 M cacodylate buffer, and then rinsed three times with deuterium-depleted water (DDW). Samples were stained with 2% uranyl acetate for 1 h, washed with distilled water, dehydrated in ethanol, and embedded in Epon EMBED 12 (EMS). The resin was polymerized at 60 °C for 24 h. The block surface was revealed with a Leica Ultracut (UC7) Ultramicrotome and analyzed with an ESEM Ouanta FEG electron microscope (Thermo Fisher Scientific, Waltham, MA, USA) with an EDS attachment.

Formalin-fixed, paraffin-embedded tissues were sectioned at 3 μm thickness and mounted on Superfrost slides. Sections were stained with hematoxylin and eosin for light microscopy. Adjacent sections were used for metallothionein immunohistochemical staining as a marker of metal-binding protein expression.

Immunostaining was performed with an automated staining system (Benchmark Ultra; Ventana Systems, Phoenix, AZ, USA). Following antigen retrieval in Tris-based buffer for 36 min at 95–100 °C, sections were incubated with rabbit anti-mouse metallothionein A1 antibody (1:200, Abcam, Cambridge, MA, USA, ab192385) for 32 min. Antigen–antibody complexes were visualized using the ultraView DAB Detection Kit (760-500, Ventana Systems) according to the manufacturer’s instructions and then amplified with the Discovery OmniMap anti-rabbit HRP system (760-4311, Ventana Systems). Sections were counterstained with hematoxylin. Immunohistochemical staining was independently evaluated by two observers, and representative sections were imaged.

For assessment of microglial activation, ionized calcium-binding adaptor molecule 1 (Iba1) immunofluorescent staining was performed on cryosections of brain tissue collected at the long-term endpoint. Sections were washed with phosphate-buffered saline (PBS), permeabilized with 1% Triton X-100 for 10 min, and blocked with 5% fetal calf serum in PBS for 60 min. Sections were then incubated overnight at 4 °C with rabbit anti-Iba1 primary antibody (1:500, Abcam, ab178846). After washing, sections were incubated at room temperature for 1 h with goat anti-rabbit IgG H&L Alexa Fluor 647 secondary antibody (1:1000, Abcam, ab150079). Nuclear counterstaining was performed with DAPI (Sigma-Aldrich, Burlington, MA, USA). Fluorescence images were acquired using an LSM 700 inverted confocal microscope (Zeiss, Oberkochen, Germany), with excitation wavelengths of 405 nm and 647 nm for DAPI and Alexa Fluor 647, respectively.

Owing to the exploratory design of the study and the limited sample sizes within specific subgroups, no formal hypothesis testing was performed. Analyses were primarily descriptive and intended to characterize distribution patterns and temporal trends. Quantitative measurements are presented as mean ± standard deviation where applicable.

## 3. Results

### 3.1. PIXE Detection in Reference Samples

**Figure 1:**
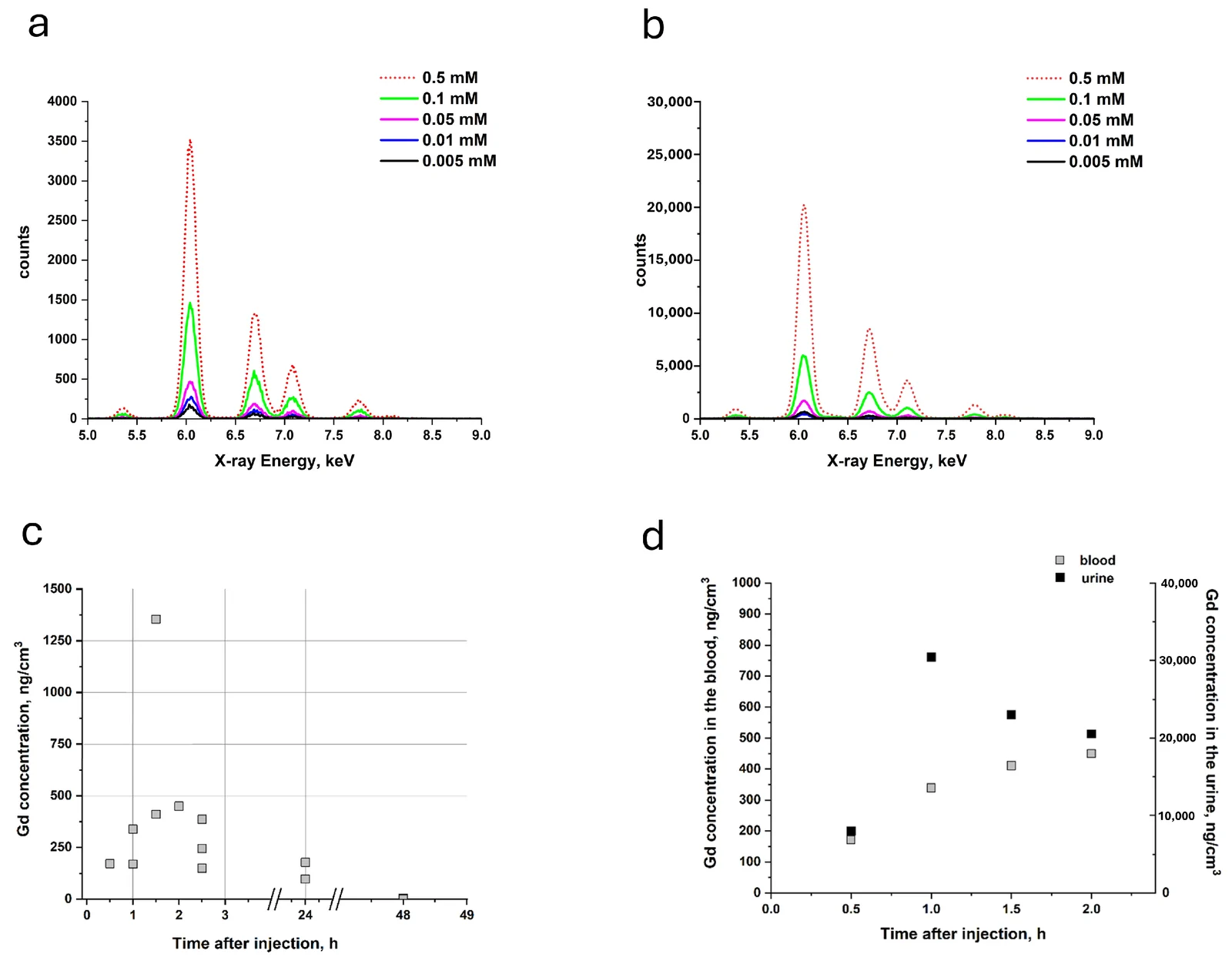
PIXE analysis of gadolinium (Gd) in reference samples and biological samples following Dotarem administration. (**a**,**b**) PIXE spectra of Dotarem (**a**) and MultiHance (**b**) reference solutions at concentrations of 0.005, 0.01, 0.05, 0.1, and 0.5 mM. Reference samples were prepared by applying 30 µL of each solution onto filter paper. Three replicates were analyzed for each concentration. (**c**) Blood Gd concentrations measured by PIXE at sampling timepoints up to 48 h following IV Dotarem administration. Breaks in the x-axis separate the early and later sampling periods. (**d**) Blood and urine Gd concentrations during the first 2 h following IV Dotarem administration. The blood measurements correspond to those shown in panel C within this interval and are displayed over an expanded time scale. Blood concentrations are plotted against the left y-axis and urine concentrations against the right y-axis.

PIXE spectra from Dotarem and MultiHance reference solutions applied to filter paper showed detectable Gd signals at the lowest tested solution concentration of 0.005 mM, based on the characteristic Lα peak (Figure 1a,b). This observation demonstrates detection capability under the reference-sample conditions but does not establish a tissue-specific detection limit.

### 3.2. Acute Biodistribution and Clearance Following Intravenous Administration

To validate systemic exposure and characterize clearance kinetics, Gd levels were assessed following IV administration of Dotarem (Figure 1c). Gd was readily detected in blood and multiple tissues immediately after injection and during the early post-injection period.

Among 21 evaluable blood samples collected within the first hours following IV injection (subset of the biodistribution cohort), measurable Gd levels were detected in 15 (Supplementary Table S1). Peak blood concentrations were observed within the first 2.5 h, followed by a progressive decline. By 24 h, Gd levels had decreased substantially, and by 48 h, Gd was no longer detectable.

Gd was detected in the urine samples collected during the first 2 h following IV Dotarem administration (Figure 1d). The plotted urine concentrations increased to the 1-h sampling timepoint and subsequently declined.

**Figure 2:**
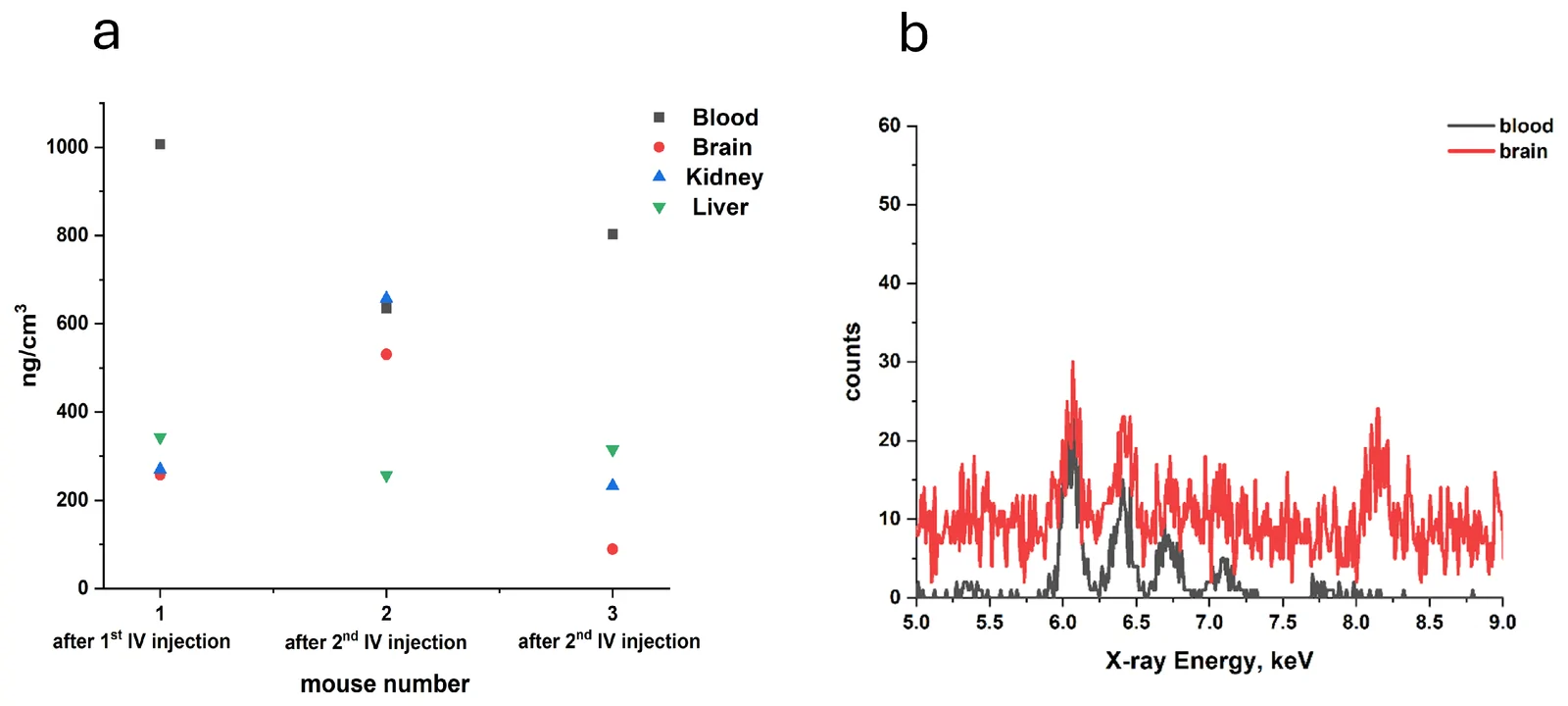
PIXE assessment of gadolinium (Gd) distribution and representative spectral findings following IV Dotarem administration. (**a**) PIXE-derived Gd measurements in blood, brain, kidney, and liver samples from three individual mice. Mouse 1 was sampled after a first injection, whereas mice 2 and 3 were sampled after a second injection. Each symbol represents a measurement from the indicated sample, and the x-axis identifies individual mice. (**b**) Representative PIXE spectra from blood (black) and brain (red) collected 24 h after a single IV Dotarem injection. The x-axis represents X-ray energy in keV, and the y-axis represents detected X-ray counts. Spectral counts illustrate the measured signals and backgrounds; quantitative Gd measurements require calibrated spectral analysis using GUPIX.

Tissue analysis using PIXE confirmed the presence of Gd in multiple organs, including brain, liver, and kidney, during the early post-injection period (Figure 2a). In brain, by 24 h, only minimal and inconsistent signals were observed (Figure 2b). Figure 2a presents PIXE-derived Gd measurements across the sampled tissues, whereas Figure 2b displays representative blood and brain spectra at 24 h; spectral peak heights alone should not be interpreted as quantitative differences in Gd concentration.

Occasional isolated Gd signals were detected in a few individual brain samples and a single tear sample, but they were not reproducible and were not associated with corresponding systemic levels. Their origin could not be confirmed, and possible explanations include analytical variability, contamination, or true localized trace amounts of Gd.

### 3.3. Electron Microscopy in the Acute Phase

**Figure 3:**
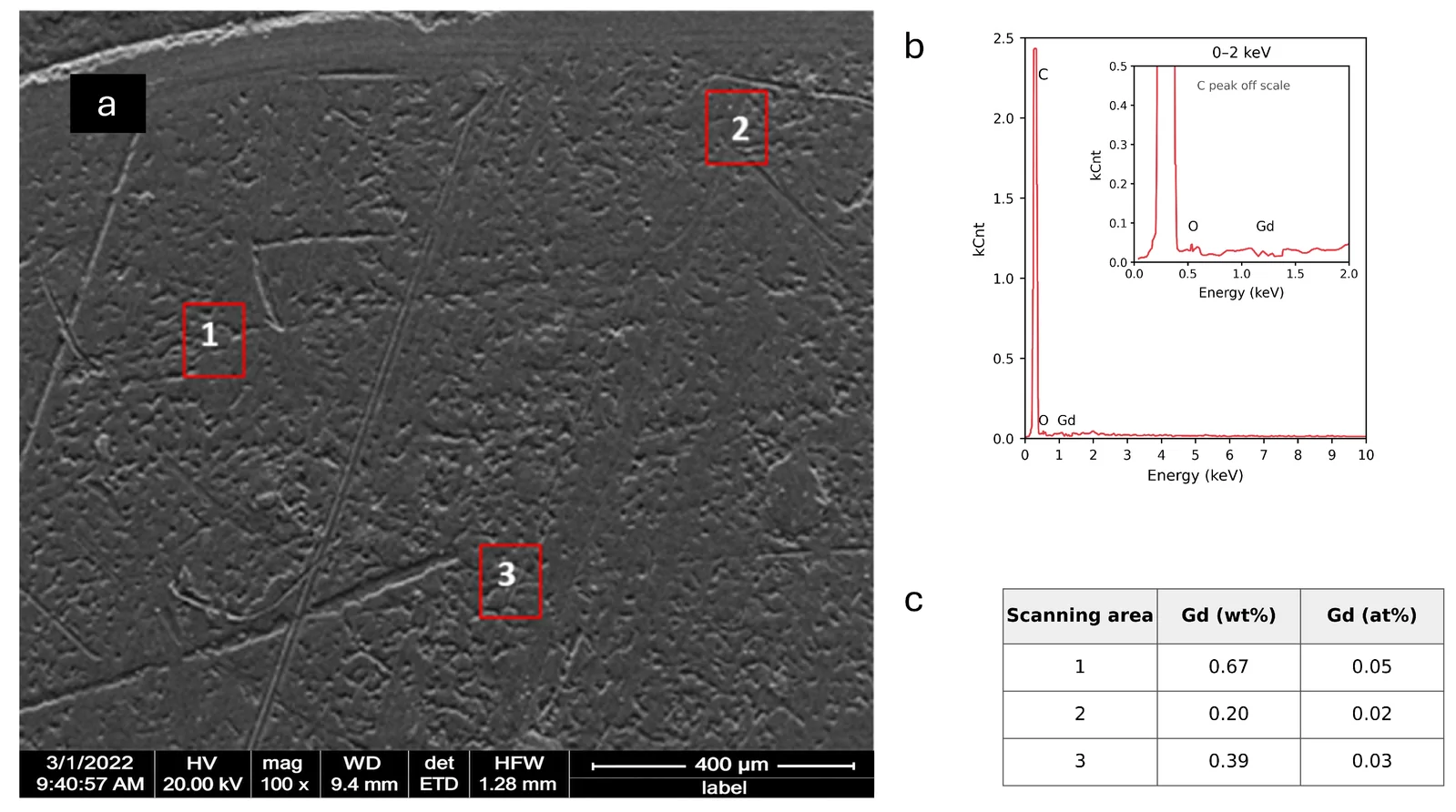
SEM-EDS assessment of selected brain tissue regions following IV Dotarem administration. (**a**) SEM image showing the three regions selected for EDS analysis. (**b**) EDS spectra obtained from these regions. The inset expands the 0–2 keV energy range; the carbon peak exceeds the displayed vertical range. (**c**) EDS-derived estimates of Gd weight percentage (Wt%) and atomic percentage (At%). Wt% represents the estimated Gd mass fraction, and At% represents the estimated Gd atomic fraction, relative to the elements included in the quantitative analysis. These values describe the selected regions rather than whole-brain Gd content.

To further assess elemental localization in the acute phase, SEM-EDS was applied to selected brain tissue samples from mice that demonstrated a Gd signal by PIXE after IV Dotarem administration. Findings were compared with IP Dotarem-treated animals and IP saline-injected controls. Analysis was limited to brain tissue harvested immediately after injection (Figure 3). EDS analysis of three selected brain regions yielded estimated Gd weight percentages of 0.20–0.67% and atomic percentages of 0.02–0.05% (Figure 3c). These localized estimates were interpreted descriptively, as a sample-specific numerical detection limit and the reliability of low-level Gd quantification were not established.

### 3.4. Long-Term Brain Retention Following Intravenous Administration

Long-term retention of Gd in brain tissue was evaluated at 28 days following a single IV injection of either Dotarem or MultiHance (*n* = 10 per group). PIXE analysis yielded no detectable Gd signal in the brain samples examined at this endpoint.

**Figure 4:**
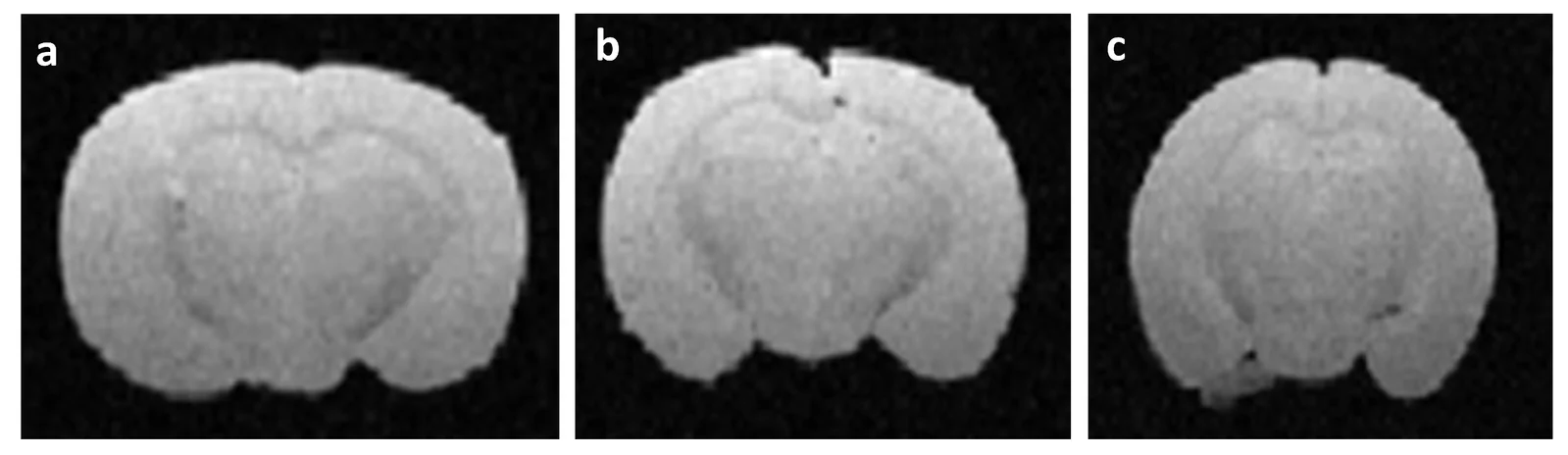
Representative ex vivo T1-weighted coronal brain MRI images obtained 28 days after a single IV administration of MultiHance (**a**) or Dotarem (**b**), and from an IP saline-injected control mouse (**c**). No apparent persistent signal changes were observed in the treated samples relative to the control. These endpoint images assess persistent signal abnormalities rather than clearance kinetics.

**Figure 5:**
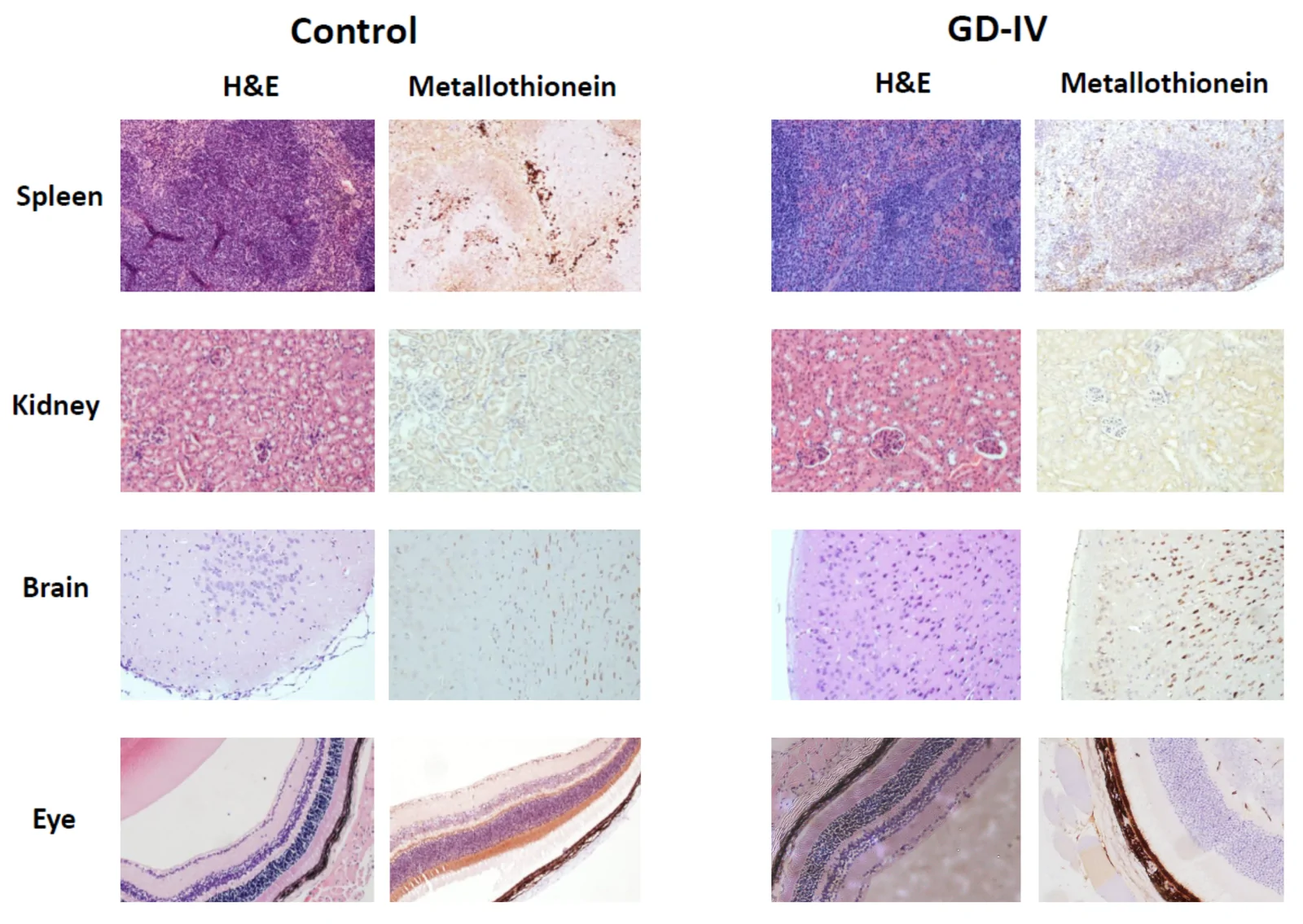
Representative H&E and metallothionein immunohistochemical staining of formalin-fixed, paraffin-embedded spleen, kidney, brain, and eye sections from mice receiving IV Dotarem or IP saline (controls). Sections were evaluated by two independent observers. H&E examination showed no overt morphological abnormalities. Metallothionein immunoreactivity is visualized as brown staining and was assessed qualitatively. The representative Dotarem-treated brain section shows more prominent immunoreactivity than the control section. Eye sections show preserved retinal layering. Scale bars indicate the dimensions shown in each panel. Magnification ×100.

**Figure 6:**
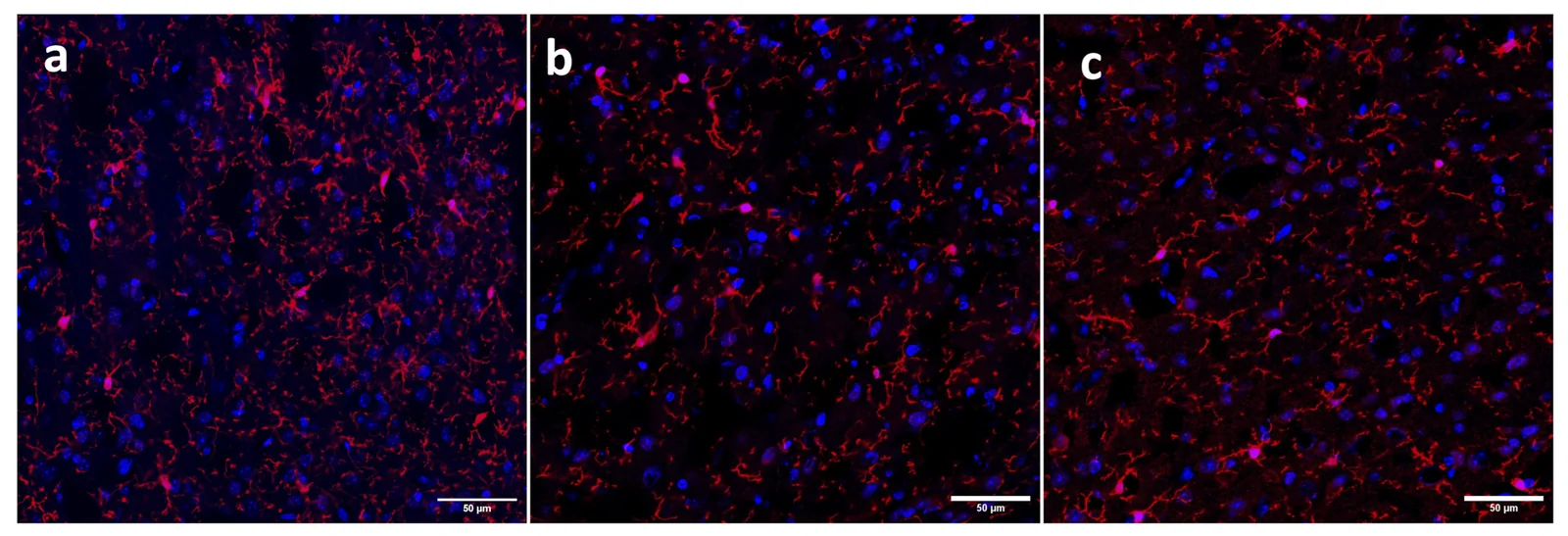
Iba1 immunofluorescence analysis (day 28) for microglial activation. Brain sections of mice injected with MultiHance (**a**) or Dotarem (**b**) and IP saline-injected control mice (**c**) showed no apparent difference in microglial activation. Red indicates Iba1 immunoreactivity in microglia, and blue indicates cell nuclei (nuclear counterstain).

At the long-term endpoint, ex vivo T1-weighted MRI showed no apparent signal changes suggestive of persistent Gd-associated enhancement compared with IP saline-injected controls (Figure 4). MRI was performed only at this endpoint and therefore did not assess clearance kinetics. PIXE analysis was negative for both contrast agents. H&E examination showed no overt morphological abnormalities in the examined sections. Metallothionein immunoreactivity varied in distribution and intensity, with more prominent staining in the representative Dotarem-treated brain section than in the control section. Representative eye sections showed preserved retinal architecture (Figure 5). Furthermore, Iba1 immunofluorescence analysis, performed at the long-term endpoint, yielded no apparent differences in microglial activation between Dotarem-treated, MultiHance-treated, and IP saline-injected control groups (Figure 6).

### 3.5. Effect of Repeated Gadolinium Exposure on Biodistribution and Retention

The repeated-IV Dotarem component comprised four subgroups: two injections 7 days apart, sampled 7 days after the last injection (*n* = 2); two injections 14 days apart, sampled 14 days after the last injection (*n* = 3); and four injections at 7–14-day intervals, sampled 30 days (*n* = 1) or 60 days (*n* = 2) after the last injection. No brain Gd signal was detected by PIXE in the samples analyzed at the 30- and 60-day endpoints. These observations are descriptive and do not establish clearance kinetics or the general absence of long-term retention following repeated exposure.

### 3.6. Assessment of Systemic Exposure Following Intraperitoneal Administration

**Figure 7:**
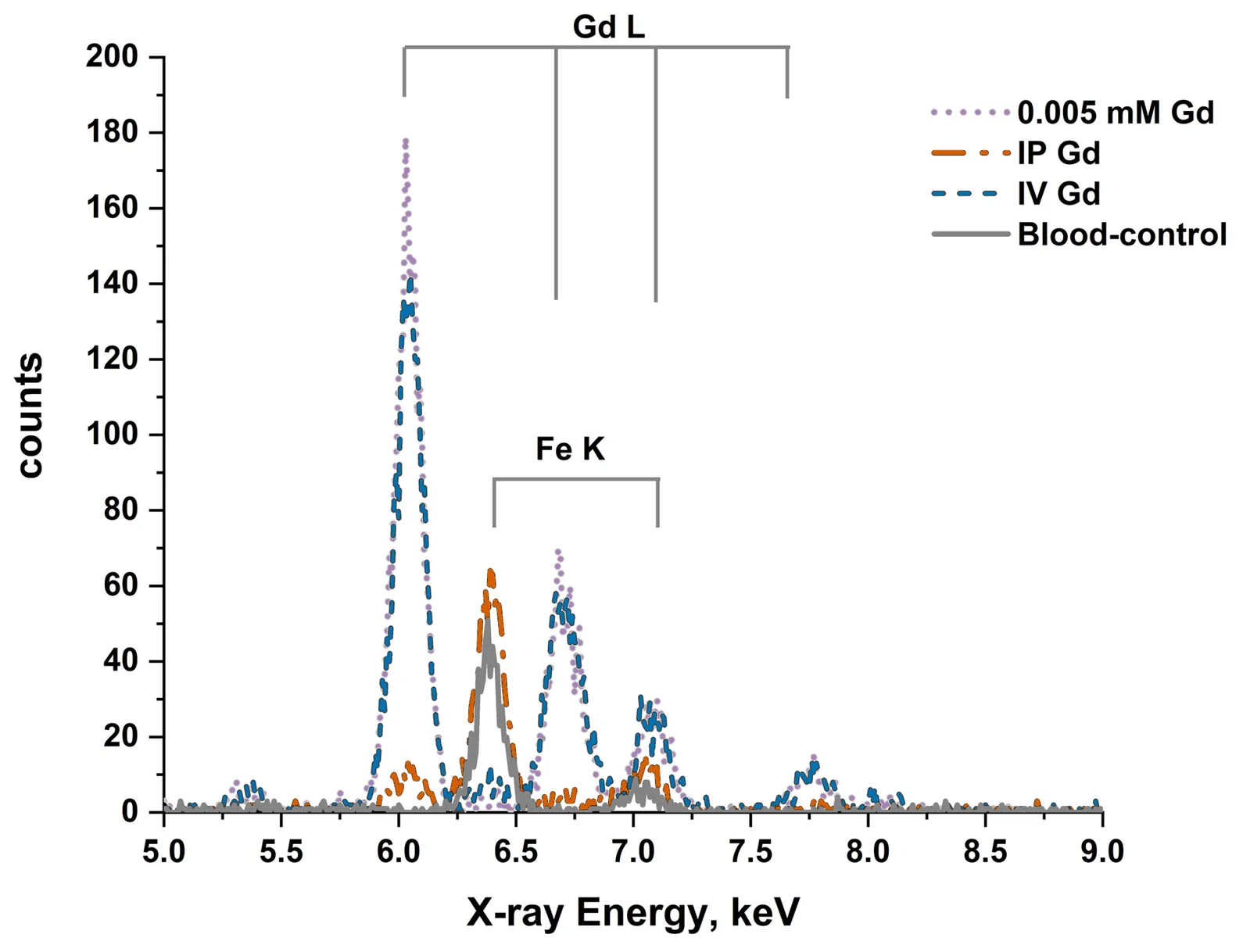
Blood PIXE spectra obtained 1 h after IV or IP Dotarem administration. Dotarem was administered at 0.5 mmol/mL, with injection volumes of 0.12 mL IV and 0.20 mL IP, corresponding to 0.06 and 0.10 mmol Gd per mouse per injection, respectively. A pre-injection blood sample and a filter-paper reference sample prepared using 0.005 mM Gd solution are included for comparison. The x-axis shows X-ray energy, and the y-axis shows detected X-ray counts. A Gd-associated signal was detected in the IV sample, whereas no corresponding signal was detected in the displayed IP samples at this timepoint.

Blood samples collected 1 h after Dotarem administration were analyzed by PIXE to assess systemic exposure following IV and IP injection. Gd was detected following IV administration, whereas no Gd-associated signal was identified in the evaluated samples following either the first or second IP injection. The repeated-IP protocols used injection intervals of 3 or 10 days (Figure 7).

Similarly, tissue analyses of brain, liver, kidney, spleen, and other sampled organs yielded no detectable Gd signal in the IP-treated tissue samples analyzed. These findings indicate that systemic Gd exposure following IP administration was not detected under the analytical and sampling conditions evaluated.

## 4. Discussion

In this study, we applied a multimodal approach to analyze Gd biodistribution, clearance, and long-term retention in a murine model following GBCA administration. Using complementary elemental, imaging, and histological methods of PIXE, MRI, SEM-EDS, and histological staining, we found no evidence of long-term detectable Gd in brain tissue at 28 days after a single high-dose IV injection. These findings remained consistent for both macrocyclic (Dotarem) and linear (MultiHance) agents.

**Figure 8:**
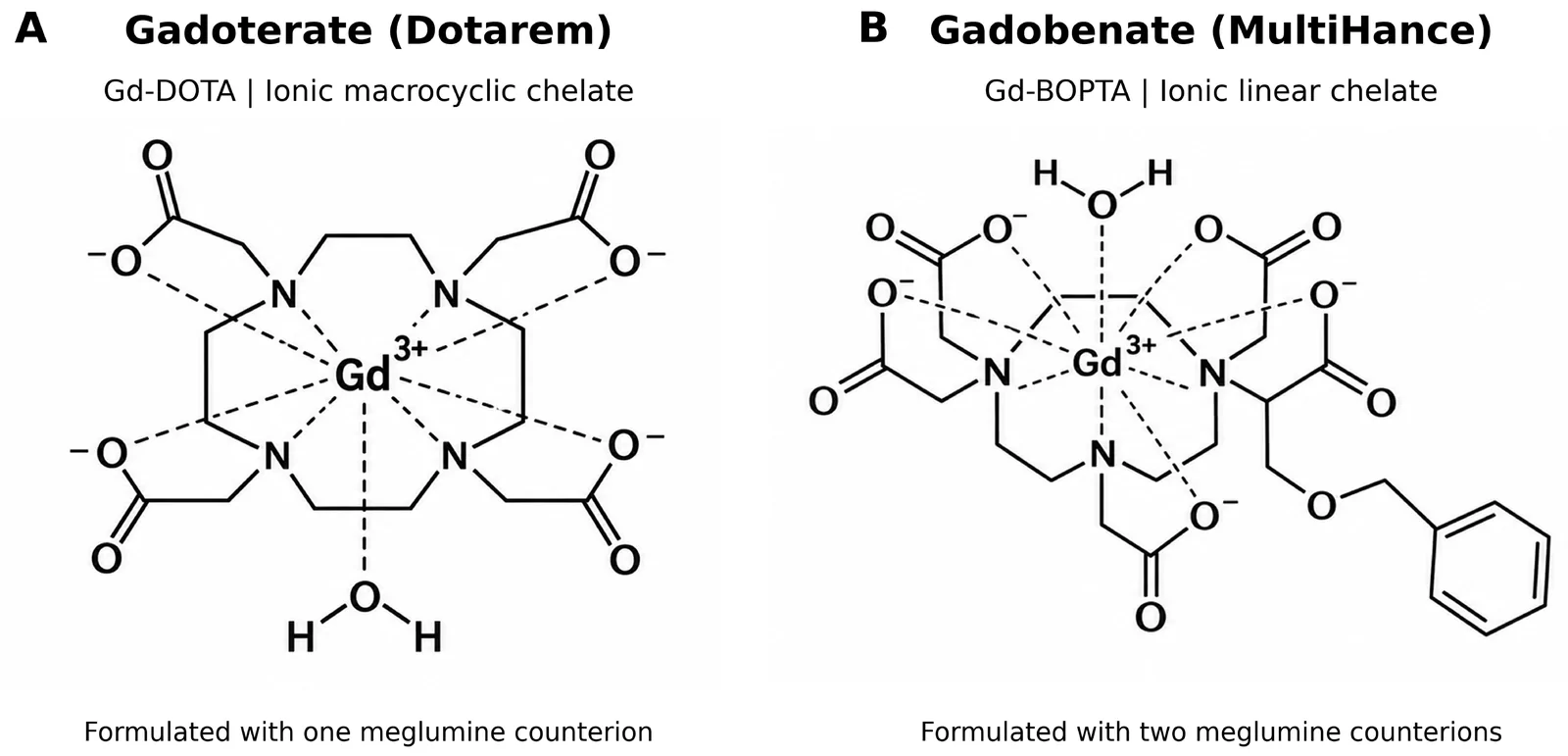
Chemical structures of the gadolinium-based contrast agents evaluated in this study. (**A**) Gadoterate (Dotarem), an ionic macrocyclic Gd-DOTA chelate. (**B**) Gadobenate (MultiHance), an ionic linear Gd-BOPTA chelate. Coordination interactions with Gd^3+^ are indicated by dashed lines. Meglumine counterions are omitted for clarity; the formulations contain one and two counterions per chelate, respectively.

The chemical structures of the two agents are shown in Figure 8. Gadoterate contains a macrocyclic DOTA ligand, whereas gadobenate contains an open-chain BOPTA ligand. Macrocyclic complexes generally exhibit greater kinetic stability and resistance to gadolinium dissociation than linear complexes, which may influence tissue retention [1,25]. Although neither agent produced detectable long-term brain gadolinium signals in the present study, this observation does not establish equivalent retention profiles or exclude differences in residual gadolinium below the detection capabilities of the methods used.

The filter-paper reference experiment demonstrated Gd detection from solutions at concentrations as low as 0.005 mM under the conditions tested. However, this assessment did not reproduce the biological tissue matrix and cannot establish a tissue-specific detection limit. Negative tissue findings therefore indicate that no Gd signal was detected under the analytical conditions used, rather than establishing the complete absence of residual Gd.

The detection of Gd in blood and multiple tissues following IV administration confirmed systemic exposure. Blood levels peaked within the first hours after injection and declined rapidly thereafter, with no detectable blood Gd signal at 48 h. This pattern is consistent with the known pharmacokinetics of GBCAs, which exhibit rapid renal elimination under normal physiological conditions [1]. In contrast, IP administration did not result in detectable systemic Gd levels on either blood or tissue analysis across repeated experiments conducted under the same sampling conditions. Therefore, subsequent analyses focused on IV administration.

Despite the substantially higher doses than those used clinically, MRI showed no apparent persistent T1-weighted signal changes at 28 days compared with IP saline-injected controls, and PIXE identified no detectable Gd signal in the examined brain samples. H&E examination showed preserved tissue morphology. Metallothionein immunoreactivity varied across the examined tissues, with more prominent staining in the representative Dotarem-treated brain section, whereas Iba1 immunofluorescence showed no apparent increase in microglial activation. These histological findings describe complementary aspects of the tissue response, including metal-associated protein expression, morphology, and microglial activation. Metallothionein responses have also been investigated in a rat liver-injury model involving gadolinium chloride [36]. However, that experimental context differs from exposure to chelated GBCAs, and metallothionein immunoreactivity alone cannot establish Gd retention. The elemental analyses, supported by the endpoint MRI findings, identified no detectable long-term brain Gd retention following single-dose exposure under the high-dose conditions tested.

The repeated-exposure component provided descriptive observations from subgroups containing 1–3 animals. No brain Gd signal was detected by PIXE in the analyzed samples at 30 and 60 days, but these small subgroups do not establish whether clearance kinetics or retention differed from those following single exposure.

Rare, isolated Gd signals were observed in a small number of brain and tear samples during the acute and intermediate phases. These signals were not reproducible and were not associated with corresponding systemic levels. Their origin could not be confirmed; possible explanations include analytical variability, contamination, or true localized trace amounts of Gd. Their lack of reproducibility limits their interpretation but does not exclude a biological origin.

Our findings can be considered alongside reports of lower brain Gd retention after macrocyclic than linear GBCA administration [17,18,19,20,21]. Previous animal studies reported variable degrees of Gd retention, particularly following repeated high-dose exposure or when using linear agents [11,18,19,20,21,22]. Regional brain Gd retention has been reported in animal studies, including in the basal ganglia and cerebellar nuclei [17,19,22]. In addition, cortical Gd retention and colocalization with parenchymal iron have been demonstrated in rats and humans [11]. Comparative studies detected residual brain Gd after macrocyclic GBCA administration, although concentrations were lower than after linear GBCA administration [17,19,20,21]. No brain Gd signal was detected by PIXE at the evaluated long-term endpoints under the analytical and sampling conditions used. These findings do not exclude residual Gd below detection capability.

Studies in humans have reported Gd retention in brain tissue following repeated exposure to GBCAs, both linear and, to a lesser extent, macrocyclic [10,11,12,13,23]. However, the clinical significance of these findings is uncertain, and histopathological confirmation is limited. Factors such as blood–brain barrier integrity, underlying disease, and cumulative exposure may influence retention in human populations. In the present study, conducted in healthy mice, there was no evidence of detectable Gd deposition.

To our knowledge, this is the first study to apply PIXE as a primary analytical tool for evaluating gadolinium biodistribution and long-term retention in brain tissue. The findings demonstrate the feasibility of integrating PIXE with MRI and histological analyses for multimodal assessment of trace-element biodistribution. PIXE provides spatially resolved elemental analysis within tissue sections [29,31], while SEM-EDS enables localized elemental assessment in selected tissue regions. These techniques provide complementary spatial information alongside bulk quantification methods. Although ICP-MS offers greater analytical sensitivity for bulk Gd quantification, conventional solution-based ICP-MS requires tissue digestion and does not preserve spatial information. The value of PIXE in this study therefore lies in its spatial characterization of elemental distribution, complemented by SEM-EDS, imaging, and histological assessment.

Several limitations of the study should be acknowledged. First, this was an exploratory study, and some subgroups, particularly those evaluating repeated GBCA exposure, included small numbers of animals. Consequently, the findings from these cohorts should be interpreted cautiously. The estimated IV doses of 2.4–3.0 mmol Gd/kg were approximately 24–30 times the standard clinical dose of 0.1 mmol/kg. This comparison describes administered doses on a body-weight basis and does not imply equivalent systemic exposure across species. The findings therefore characterize a high-dose mouse model and cannot be directly extrapolated to Gd retention or safety following standard clinical dosing. Second, although multiple tissues were evaluated, not all organs were systematically analyzed across all time points. In addition, MRI assessment was based on qualitative evaluation of ex vivo T1-weighted images and did not include quantitative techniques such as T1 mapping or signal intensity measurements, which may have increased sensitivity for detecting subtle imaging changes. Third, the maximal number of repeated injections was limited to four, whereas human exposure may involve a greater number of administrations over time. Finally, most measurements at later time points were below the detection threshold, limiting our ability to quantify very low levels of residual Gd. The origin of the few isolated positive findings remained uncertain, and analytical variability, contamination, or true localized trace amounts of Gd could not be distinguished. PIXE detection capability was assessed using reference solutions on filter paper without validation in tissues containing known Gd concentrations, limiting the interpretation of negative tissue findings. Residual Gd below the detection capability of PIXE cannot be excluded. ICP-MS or another more sensitive quantitative method was not used to corroborate the negative elemental findings; consequently, these results do not establish the complete absence of residual brain Gd.

## 5. Conclusions

Using a multimodal approach combining elemental analysis, imaging, and histology, we detected no brain Gd signal by PIXE at 28 days after a single high-dose IV administration of Dotarem or MultiHance. Gd was detectable during the acute phase, blood levels declined over time, and endpoint MRI showed no apparent persistent signal changes. The repeated-exposure observations were limited by subgroup sizes of 1–3 animals and do not support general conclusions about clearance kinetics or long-term retention following repeated administration. These observations apply to the tested analytical and sampling conditions and do not establish the complete absence of residual brain Gd. This study demonstrates the complementary value of spatially resolved PIXE analysis within a multimodal framework for evaluating Gd biodistribution and investigating tissue retention, providing a methodological framework for future studies.

## Data Availability

The datasets generated during and/or analyzed during the current study are available from the corresponding author upon reasonable request.

## Supplementary Materials

The following supporting information can be downloaded at: https://www.mdpi.com/article/10.3390/toxics14100907/s1: Table S1: Animal allocation and numbers of animals analyzed by each method, according to experimental group, injection regimen, and sampling time point. Abbreviations: IV, intravenous; IP, intraperitoneal; PIXE, particle-induced X-ray emission; MRI, magnetic resonance imaging; SEM-EDS, scanning electron microscopy with energy-dispersive X-ray spectroscopy; H&E, hematoxylin and eosin; Iba1, ionized calcium-binding adaptor molecule 1; h, hours.

## Abbreviations

The following abbreviations are used in this manuscript:

GBCA	Gadolinium-based contrast agent
Gd	Gadolinium
IP	Intraperitoneal
IV	Intravenous
MRI	Magnetic resonance imaging
PIXE	Particle-induced X-ray emission
SEM-EDS	Scanning electron microscopy with energy-dispersive spectroscopy
