# Supplementary material for: Brain Gadolinium Biodistribution, Clearance, and Retention Following High-Dose Contrast-Agent Exposure in Mice: A Multimodal Study
Source: Toxics. 2026 Oct 14;14(10):907. doi: 10.3390/toxics14100907 (PMC13709646; doi:10.3390/toxics14100907)
Supplement: Supplementary file 1 [file toxics-14-00907-s001.zip › toxics-4569853-supplementary.pdf]

Experimental component	Route	Agent	Injection regimen	Euthanasia timepoint (relative to injection)	N	PIXE n	MRI n	SEM-EDS, n	H&E, n	Metallothionein, n	Iba1, n
Biodistribution and clearance	IV	Dotarem	Single injection	0–24 h	21	21	0	5	14	14	0
				48 h	5	4	0	0	4	4	0
				21 days	4	4	0	0	4	4	0
Long-term retention	IV	Dotarem	Single injection	28 days	10	10	5	0	5	5	5
	IV	MultiHance	Single injection	28 days	10	10	5	0	5	5	5
Repeated exposure analysis	IV	Dotarem	2 injections (7 days apart)	7 days after last injection	2	2	0	0	2	2	0
			2 injections (14 days apart)	14 days after last injection	3	3	0	0	3	3	0
			4 injections (7–14 day intervals)	30 days after last injection	1	1	0	0	1	1	0
			4 injections (7–14 day intervals)	60 days after last injection	2	2	0	0	2	2	0
Route of administration	IP	Dotarem	2 injections (3-day interval)	After completion of injection protocol	7	5	0	3	5	5	0

			2 injections (10-day interval)	After completion of injection protocol	9	7	0	2	6	6	0
	IP	Saline	2 injections (3-day interval)	After completion of injection protocol	8	8	5	3	7	7	7
			2 injections (10-day interval)	After completion of injection protocol	8	8	5	3	7	7	7

Supplementary Table S1. Animal allocation and numbers of animals analyzed by each method, according to experimental group, injection regimen, and sampling time point. Abbreviations: IV, intravenous; IP, intraperitoneal; PIXE, particle-induced X-ray emission; MRI, magnetic resonance imaging; SEM-EDS, scanning electron microscopy with energy-dispersive X-ray spectroscopy; H&E, hematoxylin and eosin; Iba1, ionized calcium-binding adaptor molecule 1; h, hours.
